# Prolongierte ulzerierende Laryngitis

**DOI:** 10.1007/s00106-021-01079-0

**Published:** 2021-06-25

**Authors:** S. Reetz, M. Brockmann-Bauser, J. E. Bohlender

**Affiliations:** 1 Abteilung Phoniatrie und Klinische Logopädie, Klinik für Ohren‑, Nasen‑, Hals- und Gesichtschirurgie, UniversitätsSpital Zürich, Universität Zürich, Frauenklinikstr. 24, 8091 Zürich, Schweiz; 2 Universität Zürich, Rämistr. 71, 8006 Zürich, Schweiz

**Keywords:** Chronische Heiserkeit, Stimmlippenläsion, Laryngoskopie, Stroboskopie, Differentialdiagnosen, Chronic hoarseness, Vocal fold lesion, Laryngoscopy, Stroboscopy, Differential diagnosis

## Abstract

**Hintergrund:**

Die prolongierte ulzerierende Laryngitis ist eine seltene, gutartige, über Monate andauernde entzündliche Veränderung des Larynx. Der lupenlaryngoskopische Befund lässt einen malignen Prozess vermuten und kann somit eine Herausforderung für den behandelnden Hals-Nasen-Ohren-Arzt (HNO-Arzt) darstellen.

**Fragestellung:**

Darstellung der aktuellen Datenlage, um einen für den klinischen Alltag hilfreichen Überblick über Ätiologie, Verlauf und Therapieoptionen zu geben.

**Material und Methoden:**

Präsentation von 3 Fallbeispielen aus der Abteilung für Phoniatrie und Klinische Logopädie der ORL-Klinik des Universitätsspitals Zürich. Analyse und Diskussion der aktuellen Studienlage und von Einzelfallberichten aus der englischsprachigen Literatur.

**Ergebnisse:**

Ätiologie und prädisponierende Faktoren sind unklar. Ein vorangegangener Atemwegsinfekt mit Husten und Heiserkeit scheint die häufigste Ursache zu sein. Die Erkrankung weist einen selbstlimitierenden Krankheitsverlauf ohne strukturell dauerhafte Folgen auf. Biopsien sollten vermieden werden.

**Schlussfolgerung:**

Der typische laryngoskopische Befund zeigt umschriebene korrespondierende lanzettförmige Ulzerationen im mittleren Stimmlippendrittel. Der Krankheitsverlauf scheint selbstlimitierend zu sein und ohne strukturell dauerhafte Folgen abzulaufen. Deswegen sollten eine gute Patientenaufklärung und engmaschige laryngoskopische Kontrollen vorgenommen werden.

## Abstract (en)

**Background:**

Prolonged ulcerative laryngitis is a rare, benign inflammatory alteration of the larynx that persists for months. The laryngoscopic findings suggest a malignant process and can therefore be a challenge for the treating ear, nose and throat (ENT) physician.

**Objectives:**

Presentation of the current database to provide an overview of the etiology, progress and treatment for everyday clinical practice.

**Methods:**

Three case studies from the Department of Phoniatrics and Speech Pathology of the ENT Department, University Hospital Zurich, Switzerland, are presented. Analysis and discussion of the current literature base and of case reports in the English literature.

**Results:**

The etiology and predisposing factors for this disease are unclear. Previous respiratory infection with cough and dysphonia seems to be the most common cause. Biopsies should be avoided.

**Conclusions:**

The typical laryngoscopic findings show corresponding circumscribed lancet-shaped ulcerations in the middle third of the vocal fold. The course of the disease appears to be self-limiting and without permanent structural consequences. Therefore, good patient education and close laryngoscopic follow-up should be performed.

Die prolongierte ulzerierende Laryngitis wurde erstmals 2000 von Spiegel et al. [7, 10] beschrieben. Sie ist durch eine über Monate andauernde Heiserkeit bei gleichzeitig laryngoskopisch nachweisbaren bilateralen Stimmlippenulzerationen gekennzeichnet, die einen malignen Prozess vermuten lassen und kaum auf pharmakologische Maßnahmen ansprechen. Da HNO-Ärzte und Phoniater häufig die letzte Instanz bei prolongierter Heiserkeit sind, sind ein Überblick über die Phänomenologie sowie die Literatur zur Therapie dieses seltenen Krankheitsbildes entscheidend für eine fundierte Beratung und Behandlung.

Der Terminus Laryngitis ist zunächst eine allgemeine Bezeichnung, die einen entzündlichen Zustand des Kehlkopfes beschreibt. Die Ursachen dafür sind unterschiedlich und können multifaktoriell sein [3, 5]. Als relevante ätiologische Faktoren gelten vor allem Infektionen, die meist unspezifisch im Rahmen eines viralen Infekts der oberen Atemwege auftreten oder durch spezifische Viren, Bakterien oder auch Candidainfekte verursacht werden können. Weiterhin können laryngopharyngeale Refluxereignisse, inhalative Reizstoffe (Zigarettenrauch, Kortikoidtherapie oder Umweltnoxen), Rhinitiden (allergisch oder andere), mechanische Phonationstraumata durch Stimmmissbrauch und -überlastung [11] sowie, seltener, auch autoimmune Grunderkrankungen eine Laryngitis verursachen [7]. Der zeitliche Verlauf kann akut oder chronisch sein. Eine akute unspezifische Laryngitis weist in der Regel einen milden und selbstlimitierenden Verlauf innerhalb von 3–7 Tagen auf. Dauert dieser Krankheitszustand länger als 3 Wochen an, spricht man von einer chronischen Laryngitis [4].

Erstmals wurde in den Jahren 2000 und 2002 zum klinischen Bild der charakteristischen Erscheinungsform einer prolongierten ulzerierenden Laryngitis veröffentlicht [7, 10], die später auch idiopathische ulzerierende Laryngitis genannt wird [8, 9]. Typische Kennzeichen dieser Erkrankung sind neben einer lang anhaltenden Heiserkeit strukturelle Stimmlippenveränderungen im Form von korrespondierenden Ulzerationen, aber auch die über mehrere Monate andauernde Heilung [7].

Da gerade Patienten mit manifester prolongierter Heiserkeit Fachärzten für Hals-Nasen-Ohrenheilkunde oder Phoniatrie zugewiesen werden, soll dieser Artikel diese spezifische Form einer entzündlichen Alteration der Stimmlippen anhand von Fallbeispielen beschreiben und den Verlauf sowie die Behandlungsoptionen unter Analyse der aktuellen Literatur erläutern.

## Erstbeschreibung

Rakel et al. [7] schildert, dass vor über hundert Jahren John F. Woodward einen Artikel mit der Überschrift „Prolonged Laryngitis – Some Chief Causes and Results“ veröffentlicht und in diesem erstmals auf die Entität einer Laryngitis mit Heiserkeit, anhaltender Entzündung und vereinzelten Ulzerationen hingewiesen habe. Von 1991–1996 wurden von Spiegel und Sataloff die ersten Fälle des damals noch unbeschriebenen klinischen Krankheitsbildes einer Laryngitisform gesammelt, die durch eine länger andauernde Heiserkeit und den Nachweis von Stimmlippenulzerationen samt Beteiligung des muskulomembranösen Anteils gekennzeichnet waren. Die Entzündung dauerte über Monate hinweg an und heilte später vollständig ab im Sinne einer Restitutio [7].

In diesem Zusammenhang wurde die Bezeichnung „prolongierte ulzerierende Laryngitis“ eingeführt, die den lang anhaltenden zeitlichen Verlauf und typische Stimmlippenulzera charakterisiert. Im Gegensatz steht dazu steht der im Englischen ebenfalls verwendete Begriff einer idiopathischen ulzerierenden Laryngitis [8, 9], der die noch unklare Ätiologie berücksichtigt. In der deutschsprachigen Literatur wird teilweise sogar nur von einer ulzerierenden Laryngitis gesprochen.

## Fallbeispiele aus der Klinik

In den letzten 10 Jahren wurden in der Abteilung Phoniatrie und Klinische Logopädie in der ORL-Klinik des Universitätsspitals Zürich 13 Patienten mit einer prolongierten bzw. idiopathischen ulzerierenden Laryngitis betreut. Von diesen waren 6 Frauen im Alter von 49–83 Jahren (ø 57,6 Jahre) und 7 Patienten Männer im Alter von 34–75 Jahren (ø 46,1). Das durchschnittliche Alter betrug zum Diagnosezeitpunkt 54,3 Jahre. Neun Patienten gaben einen regelmäßigen Nikotinkonsum an.

Anhand von 3 Fällen stellen wir im Folgenden den typischen lupenlaryngostroboskopischen Befund und den Krankheitsverlauf vor.

### 1. Fall

Eine 47-jährige arbeitssuchende Patientin berichtete über eine seit 3 Monaten bestehende Heiserkeit, die in Vorstellungsgesprächen zu negativen Reaktionen seitens des potenziellen Arbeitgebers führte und sie zunehmend belastete. Anamnestisch sei ein Infekt der oberen und unteren Atemwege mit Husten vorausgegangen. Therapeutisch wurden im Vorfeld Antibiotika, Protonenpumpenhemmer, nichtsteroidale Antirheumatika verabreicht, die zu keiner Verbesserung führten. Aufgrund eines bekannten Asthma bronchiale inhalierte die Patientin regelmäßig mit Budesonid und Formoterol. Nikotinkonsum wurde verneint.

**Figure 1:**
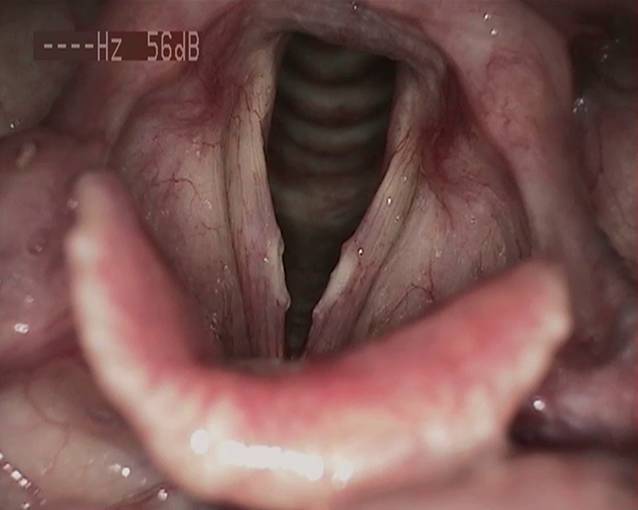

In der Lupenlaryngostroboskopie zeigten sich bei der Erstkonsultation (siehe Abb. 1) im mittleren Drittel beider Stimmlippen ulzerierende, fibrinöse Veränderungen mit verminderter Randkantenverschieblichkeit bei insgesamt geröteten epithelialen Verhältnissen der Stimmlippen. Die Sprechstimme klang auditiv perzeptiv hochgradig heiser (G3R3B3A2S2).

**Figure 2:**
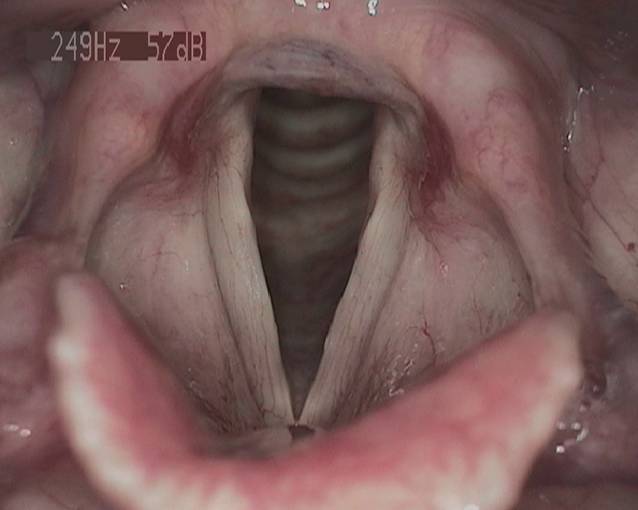

Wir initiierten eine inhalative Therapie mittels Macholdt-Inhalator mit einer klinikinternen Lösung (Tab. 1) mit Kortison für 2 Wochen und im Anschluss ohne Kortison für weitere 3 Wochen. Klinische Kontrollen fanden in 4–6 wöchentlichen Abständen statt. Insgesamt dauerte der Verlauf bis zur vollständigen Remission weitere 4 Monate an, sodass die Gesamtdauer der Erkrankung 7 Monate betrug. Der lupenlaryngostroboskopische Befund in der Abschlusskontrolle zeigte bis auf eine äußerst diskrete Verdickung im mittleren Stimmlippendrittel links vollständig abgeheilte Verhältnisse bei auditiv-perzeptiver noch leichtgradig rauer Stimmqualität (G1R1B0A0S0; Abb. 2).

**Table 1:** 

Ohne Kortison	Mit Kortison
Panthenol NAS ratiopharm 50 ml 3,0 %ige NaCl-Lösung ad 100 g	Panthenol 50 ml Prednisolut ex amp. 50 mg 3,0 %ige NaCl-Lösung ad 100 g
M.f. solutio zur Inhalation Aseptische Bereitung Abfüllung in 2 × 50 g Dosierbechern	M.f. solutio zur Inhalation Aseptische Bereitung Abfüllung in 2 × 50 g Dosierbechern

*m.f. solutio* mische, dass es eine Lösung ist; *amp.* Ampulle

### 2. Fall

Eine 50-jährige Patientin mit persistierender Heiserkeit, Trockenheits- und Globusgefühl im Hals seit 2 Monaten ohne vorausgehenden Infekt der oberen Atemwege wurde uns zugewiesen. Eine Therapie mit Protonenpumpenhemmern war extern verordnet worden und blieb ohne subjektive Verbesserung. Es bestand ein regelmäßiger Nikotinkonsum über mehrere Jahre bei ansonsten gesunder Patientin.

**Figure 3:**
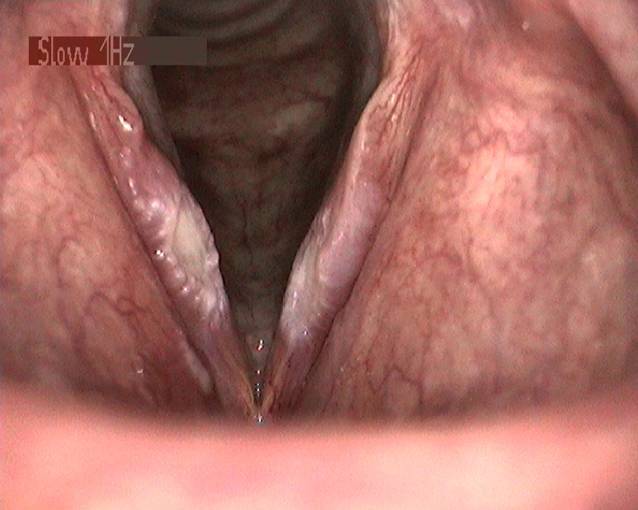

**Figure 4:**
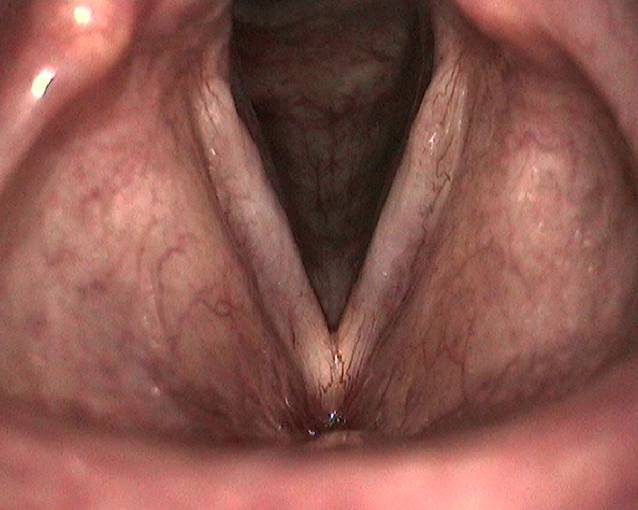

In der Lupenlaryngostroboskopie zeigten sich bei der Erstkonsultation (Abb. 3) im mittleren Drittel beider Stimmlippen korrespondierende lanzettförmige, scharf abgrenzbare Ulzerationen bei ansonsten reizlosen laryngealen Verhältnissen. Es waren keine Randkantenverschiebungen oder Amplituden nachweisbar. Die Sprechstimme klang auditiv perzeptiv hochgradig heiser (G3R3B3A2S3). Es wurden eine Inhalationstherapie mittels Macholdt-Inhalator mit einer klinikinternen Lösung (Tab. 1) mit Kortison für 3 Tage und ohne Kortison für weitere 4 Wochen rezeptiert, sowie 6 Sitzungen logopädische Stimmtherapie zur Vorbeugung einer Fehlkompensation eingeleitet. Nach 3 Monaten ließ sich neben der perzeptiven Stimmverbesserung (G1R1B0A0S1) endoskopisch eine vollständige Remission nachweisen (Abb. 4).

### 3. Fall

Es erfolgte die Zuweisung eines 75-jährigen Patienten mit anhaltender Heiserkeit seit 4 Wochen nach einem Infekt der oberen Atemwege. Vorgängig war eine perorale Therapie mit Steroiden eingeleitet worden ohne wesentliche subjektive Verbesserung. Nebendiagnostisch bekannt waren ein Asthma bronchiale mit notwendiger regelmäßiger Inhalation mit Budesonid und Formoterol und eine arterielle Hypertonie. Ferner bestand ein regelmäßiger Nikotinkonsum.

**Figure 5:**
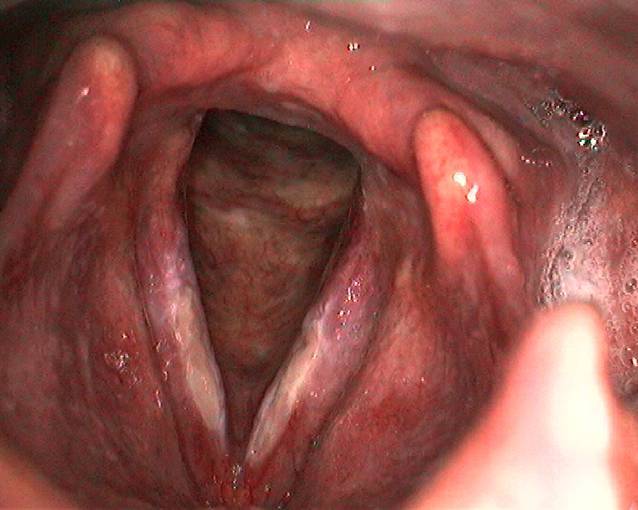

In der Lupenlaryngostroboskopie waren umschriebene lanzettförmige Ulzerationen auf den Stimmlippen korrespondierend im mittleren Drittel nachweisbar bei insgesamt geröteten laryngealen Schleimhautverhältnissen (Abb. 5). Die Randkantenverschieblichkeit und die Amplitude waren im Bereich beider Stimmlippen reduziert. Die Sprechstimme war auditiv perzeptiv hochgradig heiser (G3R3B3A2S2).

**Figure 6:**
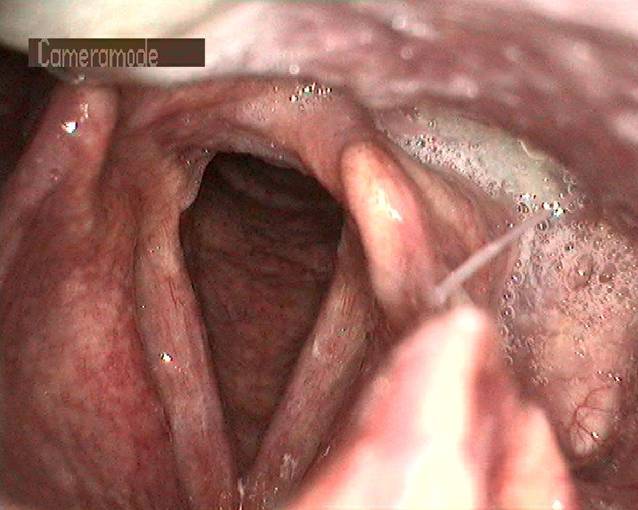

Wir verordneten eine perorale Antibiotikatherapie und eine Inhalationstherapie mit einer klinikinternen Lösung (Tab. 1) mit Kortison für 1 Woche und ohne Kortison für 4 Wochen mit dem Macholdt-Inhalator und empfahlen zudem Stimmschonung. Nach 3 Monaten waren eine vollständige Abheilung nachweisbar und der Patient subjektiv beschwerdefrei. Auditiv perzeptiv klang die Sprechstimme noch leichtgradig rau und behaucht (G1R1B1A0S0; Abb. 6).

## Diskussion der aktuellen Literatur

Seit der Erstbeschreibung der prolongierten bzw. idiopathischen ulzerierenden Laryngitis im Jahr 2000 gibt es nur wenige Erfahrungsberichte und Studien in der englischsprachigen Literatur. Die erste Beschreibung eines Kollektivs von 14 Patienten mit ulzerierender Laryngitis wurden von Rakel et al. [7] im Jahr 2002 veröffentlicht. 2011 wurden durch Hsiao et al. 2011 [5] eine Beobachtungsstudie mit 33 Patienten und durch Simpson et al. 2011 [8] eine multiinstitutionelle Erhebung von 15 Patienten umgesetzt. Die letzte Studie mit 23 Patienten wurde von Young et al. 2018 [13] beschrieben und schloss neben den endoskopischen Befunden auch die Auswertung des Voice-Handicap-Index (VHI-10) mit ein. Diese Studien sowie 3 Fallberichte [1, 9, 12] bilden nach unserem Wissensstand den in Tab. 2 aufgelisteten aktuellen Bestand an Literatur über die prolongierte/idiopathische ulzerierende Laryngitis.

**Table 2:** 

Literatur	Patienten (*n*)	Alter (Jahre)	Noxen	Beruf mit hoher Stimmbelastung	Nebendiagnosen	Krankheitsdauer	Therapie
Rakel et al. 2002 [7]	14 (m:9/w:5)	23–65 (ø: 38,4)	Nikotin: 2 Alkohol: 3	12	Asthma: 6 Allergie: 5 Fieberbläschen: 3 Depression: 2	?–3 Jahre	Steroide p.o., Antibiotika, PPI, Stimmruhe, Stimmtherapie, Biopsie (2/14)
Hsiao et al. 2011 [5]	33 (m:13/w:20), Rezidiv 5 Patienten	26–76 (ø: 49,5)	Nikotin: 4 Alkohol: 3	11	Asthma: 2	4–20 Wo (ø: 9,4)	Beobachtung, unspezifische Therapie
Simpson et al. 2011 [8]	15 (m:1/w:14), Rezidiv 2 Patienten	33–72 (ø: 49)	Nikotin: 0	Nicht bekannt	Asthma: 6	2–10 Mo (ø: 3,3)	Antimykotika: 14 (93 %), PPI 13 (87 %), Steroide p.o.:4 (27 %), Antibiotika 2 (13 %)
Young et al. 2018 [13]	23 (m:7/w:16)	26–79 (ø: 49)	Nikotin:39 %	52 %	Keine Angaben	1–36 Mo (ø: 6,8)	PPI: 85 %, Antibiotika 22 %, Steroide p.o. 52 %, Virostatikum 52 %, Antimykotika 39 %, Stimmruhe 65 %
Sinclair et al. 2013 [9]	1 m	55	Früher Nikotinkonsu	Nicht bekannt	Diabetes mellitus II GERD	3 Mo	Steroide p.o., Antibiotika
Toland et al. 2013 [12]	1 w	18	Nicht bekannt	Ja	Keine	3 Mo	Steroid p.o., PPI, Antimykotika, Stimmruhe
Beaver et al. 2005 [1]	1 w	57	Keine	Nicht bekannt	Nicht bekannt	2,5 Mo	Antibiotika, Montelukast, Kortison-Nasenspray, Steroide p.o.

*m* männlich, *w* weiblich, *ø* Durchschnitt, *GERD* Gastroösophageale Refluxkrankheit, *Wo* Wochen, *Mo* Monate, *p.o.* per oral, *PPI* Protonenpumpenhemmer

### Ätiologie

Zusammenfassend gesehen ist die genaue Ätiologie für dieses spezifische Erscheinungsbild einer Laryngitis weiterhin unbekannt. Simpson et al. [8] postulierte unter anderem eine mögliche Herpes-simplex-Virus-Infektion als Ursache, da er wenige Fälle von Rezidiven nach Monaten und Jahren beobachtete. Rückblickend begann die Erkrankung häufig nach einer vorangegangenen Atemwegsinfektion, die mit schwerem Husten und Heiserkeit einherging. Weiterhin zeigte sich ein über viele Monate andauernder Verlauf [5, 7, 8, 13]. Geschlecht und Alter scheinen kein wesentlicher prädisponierender Faktor für die Erkrankung zu sein, obwohl Rakel et al. 2002 [7] einzig einen größeren Anteil an Männern mit einschloss und die Altersspanne um circa 10 Jahren unterhalb der übrigen Studien lag [5]. In der Zusammenschau aller spezifischen Veröffentlichungen (Tab. 2) trat die Erkrankung jedoch am häufigsten bei Frauen im 4. und 5. Lebensjahrzehnt auf [8]. Ein signifikanter Zusammenhang mit Nikotinkonsum oder Begleiterkrankungen, wie z. B. Asthma bronchiale, Diabetes mellitus Typ II oder gastroösophagealer Reflux, ließ sich bisher nicht signifikant nachweisen.

### Behandlung und Diskussion weiterführender Untersuchungen

In den meisten Studien und Fallberichten [1, 7–9, 12, 13] wurden die Patienten individuell symptomatisch medikamentös behandelt, darunter waren Steroide, Antibiotika, Antimykotika und Protonenpumpenhemmer am häufigsten vertreten [1, 7, 8, 12, 13]. Ergänzend wurden stimmhygienische Maßnahmen wie Stimmruhe oder sogar Stimmtherapie empfohlen [7, 12, 13]. Eine spezifische Kausaltherapie ist bisher aber nicht bekannt. Eine kürzlich veröffentlichte Studie [6], die laryngologisch tätige Hals-Nasen-Ohren-Ärzten in den USA befragte, zeigte, dass das Management der prolongierten ulzerierenden Laryngitis weiterhin im klinischen Alltag eine Herausforderung für den behandelnden Arzt darstellt.

Von einigen Autoren wird sogar angenommen, dass es sich um eine selbstlimitierende Krankheit handelt und ein spontaner Heilungsverlauf in der Regel zu erwarten ist [5].

Die laryngoskopische Diagnostik stellt unabhängig von den Symptomen eine Herausforderung für den behandelnden HNO Arzt dar. Bei flexibler Endoskopie mit geringer Auflösung besteht die Gefahr, dass ein maligner Prozess klassifiziert wird und voreilig eine Biopsie oder mikrochirurgische Exzision erfolgt. In den aufgeführten Studien wird die Indikation einer Biopsie bei diesem scheinbar suspekt malignen Befund kritisch beurteilt. Nur bei hochgradigem Verdacht auf eine bösartige Erkrankung sollte eine Biopsie erfolgen, um dauerhafte Veränderungen und Vernarbungen der Stimmlippen bei einer prolongierten ulzerierenden Laryngitis zu verhindern [5, 7, 8].

Wird in Zusammenschau der erhobenen Befunde und Anamnese die Diagnose einer prolongierten ulzerierenden Laryngitis gestellt, sollte der Patient detailliert aufgeklärt werden, insbesondere im Hinblick auf die Dauer der Erkrankung, auf engmaschige Kontrollen und potenzielle Behandlungsoptionen. Aspekte einer stimmbezogenen eingeschränkten Lebensqualität im Berufs- und Sozialleben, wie in unserem ersten Fallbericht, sollten berücksichtigt werden. Ein umfassend aufgeklärter Patient kann in der Regel mit seiner Erkrankung deutlich besser umgehen [2].

## Fazit für die Praxis

- Typische laryngoskopische Befunde für Patienten mit prolongierter ulzerierender Laryngitis sind umschriebene korrespondierende lanzettförmige Ulzerationen im mittleren Stimmlippendrittel.
- Über Monate andauernder und selbstlimitierender Krankheitsverlauf ohne strukturell dauerhafte Folgen bei unklarer Ätiologie.
- Biopsien sollten auch bei scheinbar suspektem Befund vermieden werden.
- Angemessene detaillierte Aufklärung und regelmäßige laryngoskopische Kontrollen des betroffenen Patienten werden empfohlen.
- Individuell symptomatisch angepasste Therapieoptionen sind z. B. Antibiotika, PPI (Protonenpumpenhemmer), Steroide, Stimmtherapie bei insgesamt unklarem kausalem therapeutischem Erfolg.
